# Observations on Dr. Harrison's Bill

**Published:** 1811-05

**Authors:** 


### *To the Editors of the Medical and Physical Journal.*

GENTLEMEN,

HAVING once presented myself to your notice as the friend of a gradual, mild, and practicable reformation of medical abuses, in answer to the unfounded objections which I had heard in company, and met with in reading various periodical works, I fancy myself called upon to defend the consistency and propriety of my former remarks; I shall therefore reply in few words to the rather uncourteous attack of your correspondent A. H. in the February number. My object being to obtain what I deem reasonable to all parties, in the first instance, and to provide for effectual correction by slower means, I shall endeavour to conduct myself with a temper and moderation befitting the occasion, and that distrust of myself which is due to a complex and involved question. Various hasty and crude objections having been made both to the *language and matter* of the bill, by different writers, I endeavoured to obviate *verbal criticism*, by stating that the bill was drawn out by a Barrister of leading eminence. For the *instructions* of course he cannot be answerable; these were given to him by others, and I distinctly noticed both parts of the argument in the following terms. "Should any person remain sceptical after this explanation, he will, I think, do well to state his difficulties, and have the sanction of some legal practitioner, before he ventures hastily and inconsiderately to oppose and condemn clauses, which have been framed by a Barrister of the first eminence in this department of the law, and have been unequivocally approved by some of the most respectable physicians, surgeons, apothecaries, and accoucheurs of the present age."

First.—As to the registry, we are gravely told by A. H. that it is provided that "all physicians, surgeons, &c. shall enter their names in one grand register, for which they shall pay a certain sum, and place their names upon the doors of their respective dwellings." Now my knowledge of the bill enables me to assert, that the present generation will not be called upon for a single sous; they are to be merely registered. Indeed, the term "*to enter*"* into the profession im-

* See Dr. Harrison's address to the Lincolnshire Ben. Med. Soc. 111.

plies plies no more. It is true that their successors, *i. e.* such as *shall not have entered* their names on the first register, will have to pay twenty pounds to be employed for medical purposes mentioned in the bill itself. The clause is not retrospective.

Secondly.—"The powers of all existing corporations shall be confirmed." Now, really Gentlemen, I had always conceived one excellency of the bill to consist in its studiously passing over all disputed points. As far as my limited judgment enables me to decide, the distressing misunderstandings in the profession relate wholly to the bye-laws, and these are left unnoticed. To me it does not appear that the stability of the colleges will be either improved or weakened, in the first instance, by the bill itself. The Licentiates of the College of Physicians may prefer their claims with as much *force* and *cogency*, after, as before the passing of the Bill, as may the commonalty of surgeons and independent apothecaries, for the severe treatment received from their respective corporations. The surgeons and apothecaries excluded from all participation in the honors and emoluments of their respective bodies, will therefore possess a similar feeling to the physicians, and may be expected to unite their joint endeavours for the same laudable purpose. In proportion, therefore, as the list of licensed physicians, surgeons, and apothecaries, is extended in the counties and boroughs, their joint interest will be proportionally increased in the legislature; while that of the Fellows of the Royal College of Physicians, of the Examiners in the College of Surgeons, and Rulers in the Apothecaries Company, will obtain no new political strength. I firmly believe the privileged members of the corporate bodies have *anticipated* this difficulty among others, and are therefore determined to oppose themselves to every scheme of medical reform. However mild the present Bill may appear to A. H. it is objected to for its *oppressive tendency* by the colleges, and so violently, that I am given to understand, the legislative friends of the Bill have been led to *hesitate* about ushering the measure into parliament until the storm is dispersed. How it will end, for the present, is at this moment extremely doubtful. Should the colleges acquiesce, I conceive the plan will be carried into effect this session, otherwise it must be deferred to a more favourable period, when the public, and the independent faculty, shall have been induced to take a more lively concern in the improvement of medical science and practice. If the *great body of the faculty chuse to come forward in defence of their injured rights*, their united efforts will be irresistible. The Fellows of the College of Physicians, and Courts of Examiners,

 and and of Assistants in the College of Surgeons amount only to about 70 persons, while the remaining practitioners in England and Wales are estimated at 15,000. A contention between the two parties must, as it ought, be unfavourable to the former.

Thirdly, "That the magistrates at the quarter-sessions shall be authorized to license every ignorant pretender to medical skill who thinks fit to apply." Here, too, I am at issue with your correspondent. According to my conception of the Bill, the magistrates are only empowered to license *such as are really in practice* when the act shall be obtained, and in the way "in which they shall have formerly acted."* They cannot accept or sanction future candidates. Their authority being limited and confined to the present establishment, empirical practice must terminate with this generation. Whatever sentiments we may entertain of the matter, it cannot be denied that irregular pretenders have so many friends in the higher orders of society, that no bill would be passed by the legislature the object of which went to an immediate extinction of quacks. All that can be safely attempted is to secure the suppression of notorious offenders, and provide for the termination of the remainder with their natural lives, or at the will of the magisterial bench. Suppose the magistrate to disallow only nine in ten at their first meeting, much good will immediately result to the faculty as well as to the society at large from the diminution, because a greater number of the sick must thereafter apply to the regular fraternity, and their disorders being, I trust, better understood, would be more skilfully treated.

Fourthly, "The sale of stamped medicines shall be countenanced and legalized *in omnia secula seculorum*. Here again your correspondent and I entertain very different opinions. In my judgment the Bill neither affords additional "countenance, nor legalizes the sale of stamped medicines." They are left in statu quo. They are at present sanctioned by the legislature, and the bill merely declares that it does not "extend to prevent or hinder any person or persons from selling any drugs or medicines on the sale of which any stamp duty is by law imposed, in like manner as they might have heretofore done." It would be folly in these times to try to oppose the stream of popular prejudice, and therefore the contest is prudently avoided. The gentlemen of the Law deemed it necessary to insert the clause in question, to shew that no interference was intended with the present law relative to quack medicines, and of course their advice was admitted. Having briefly noticed, and I trust fully defended the Bill

* See Dr. Harrison's Address, p. 110.

from from the four objections stated above, I have to observe that to my knowledge *it did include other clauses*, which on the recommendation of parliamentary advisers, were omitted in the printed sketch. It was by them judged most prudent to avoid whatever might *irritate the corporate bodies*, from a belief that with their approbation the bill would experience *no difficulties* in its progress through parliament. Notwithstanding this courtesy shewn to the colleges, it is proved by their obstinacy that no friendly communication can subsist between them and the partisans of the Bill. They will have no connection with *alieni homines* as they are pleased to call the friends of reform. The framers of the bill had to consider and provide for three distinct parties, whose prejudices, passions, and interests, unhappily for mankind, are openly opposed to each other, viz. the public, the corporate bodies, and independent practitioners. In some few points they have similar feelings, and may therefore be expected to unite their cordial exertions. To catch and act upon general views has always been a primary consideration with the active partisans; for they well knew that by dividing the force they should weaken the efforts, and probably frustrate the whole plan. Under this impression they limited their endeavour to *the gradual amendment of medical men, by filling up vacancies as they occur in the regular establishment* with confidential and respectable practitioners. Such is the cardinal hinge, the fundamental object of all their deliberations, and to which the machinery of the bill is made subservient. This, it is true, is slow, gradual, and to the consideration offers a distant mode of obtaining the end. It does not, therefore, accord with the zeal and impatience of sanguine tempers. With them every thing is to be attempted immediately and at once. Nothing must be left for others to achieve. Were the whole attainable and within grasp, I should indeed blame the associated faculty for substituting a timid policy to a bold enterprize. Under existing difficulties, without a *medical representative in parliament*, we must rely wholly on the justice and obvious tendency of our complaints. Nothing involved, nothing doubtful, nothing concealed, should be hazarded. By attempting too much we shall lose all: by carrying on the proceedings with deliberation, we may ultimately obtain every thing. Nor is the end so remote as many suppose. The present generation will soon pass away, and a new one be substituted. Suppose the medical establishment of England and Wales to be 15,000, under the various denominations of Physicians, Surgeons, Apothecaries, and Venders of Drugs. The aggregate lives may, I conceive, be individually worth about seven years' purchase. According to this estimate 2000 new practitioners ners will be admitted the first year. Every succeeding year will increase the number. These having been regularly trained and fitted for their momentous duties will act towards their seniors and employers with greater propriety, than we now experience from each other. They will not be led to practise the low arts of the half informed and half instructed, who are the assassins of professional respectability, and of every thing commendable in the art. If persons can at pleasure call themselves Physicians, Surgeons, Apothecaries, and Druggists, without undergoing a competent measure of study, and without possessing a diploma or indentures of apprenticeship, surely some check is wanted to stop the supply of these dangerous fellow labourers. That such things can and are daily committed, no observer will venture to deny. Arrest this torrent *for five years only*, and I dare hazard an opinion, that the face of things will in that short time be much mended. Lord Kenyon's Bill, as it is called, has not, I think, operated more than ten years, and its effects upon the legal profession are strikingly obvious. The Attornies are already become a different race of men, though many of them commenced their career as humbly as any in medicine, viz. in the two-fold capacity of apprentice and stable-boy, or house-servant. The increased respectability of the young attorney is communicated to the senior members, and general professional character is daily improving, equally to the benefit of the Law and the Clients. Such would, I doubt not, be the pleasing result by securing a better education and preliminary examinations as provided in the bill. Let these improvements be suffered to operate till circumstances will admit of an additional bill. This again may be followed by others. Thus, by zealous co-operation and vigorous exertions, a full and complete reformation may at length be obtained. Such is the prudent, and as it appears to me, the only safe way of proceeding, in a complicated undertaking where so many interests require to be reconciled, and in an *assembly too, where we have no professional brother to defend our cause.*

